# Improved understanding of aqueous solubility modeling through topological data analysis

**DOI:** 10.1186/s13321-018-0308-5

**Published:** 2018-11-20

**Authors:** Mariam Pirashvili, Lee Steinberg, Francisco Belchi Guillamon, Mahesan Niranjan, Jeremy G. Frey, Jacek Brodzki

**Affiliations:** 1 Mathematical Sciences, University of Southampton, Southampton, UK; 2 Department of Chemistry, University of Southampton, Southampton, UK; 3 ECS, University of Southampton, Southampton, UK; 4 Institut de Robòtica i Informàtica industrial, CSIC-UPC, Llorens i Artigas 4-6, 08028 Barcelona, Spain

**Keywords:** Solubility, Chemical space, Mapper, Persistent homology

## Abstract

Topological data analysis is a family of recent mathematical techniques seeking to understand the ‘shape’ of data, and has been used to understand the structure of the descriptor space produced from a standard chemical informatics software from the point of view of solubility. We have used the mapper algorithm, a TDA method that creates low-dimensional representations of data, to create a network visualization of the solubility space. While descriptors with clear chemical implications are prominent features in this space, reflecting their importance to the chemical properties, an unexpected and interesting correlation between chlorine content and rings and their implication for solubility prediction is revealed. A parallel representation of the chemical space was generated using persistent homology applied to molecular graphs. Links between this chemical space and the descriptor space were shown to be in agreement with chemical heuristics. The use of persistent homology on molecular graphs, extended by the use of norms on the associated persistence landscapes allow the conversion of discrete shape descriptors to continuous ones, and a perspective of the application of these descriptors to quantitative structure property relations is presented.

**Electronic supplementary material:**

The online version of this article (10.1186/s13321-018-0308-5) contains supplementary material, which is available to authorized users.

## Introduction

It is estimated that approximately 40% of all drug discovery programs are abandoned due to problems with bioactivity [1], with solubility being a major concern for oral delivery.

While the field of solubility prediction is well-established, with several standard data sets and models being produced to generate predictive models, it is however, widely accepted that such models are inherently flawed, due to experimental difficulties resulting in significant uncertainties in measuring solubility estimated to be around 0.6 log units [2]^1^.

Prediction algorithms that followed after [2] usually did not take this into account and therefore overfit, with a residual error lower than the estimated experimental uncertainty.

Huuskonen’s papers [3, 4], written two years prior, in 2000, used multilinear regression and artificial neural networks on 30 electronic and topological features, and was able to achieve an $r^2$ value of 0.86, with a standard deviation of 0.5 log units.

In 2004 [5], Delaney used linear regression on 9 features, subsequently reduced to 4, to predict solubility. The improvement in accuracy between Huuskonen and Delaney’s work falls within the error range of experimental solubility values.

The same issue arises in the interpretation of the 2013 results by Baldi et al. [6], which uses novel methodologies, utilising the connection between recursive deep learning architectures and the molecular graph, but does not alter the problem statement: it once again tries to improve the prediction of solubility, acknowledging that the improvement falls within the expected experimental uncertainties.

In 2009, the Solubility Challenge was designed to assess the state of the field [7]. This consisted of modeling the solubility of relatively few, but highly trusted measurements. The results of the challenge were mixed, with $r^2$ values ranging from 0.02 to 0.65 for entrants.

One of these entrants, Hewitt et al. constructed models of various complexities including linear regressions and neural networks [8]. They found that the multi-linear regression model outperformed the more complex counterparts, although it was suggested that this may be due to the limited number of molecules available in the solubility challenge data set.

Palmer et al. showed that even if we were able to produce high-quality experimental data, the deficiencies in quantitative structure property relations (QSPR) models (be that the algorithms themselves, or incomplete descriptor sets), lead to inaccuracies in solubility prediction [9]. It is clear that new descriptors are required, alongside high-quality data sets. Therefore, rather than an incremental improvement of precision, we should shift to a deeper understanding of what is determining molecular solubility, and the chemical properties of the data. In particular, the main use of current solubility prediction tools should at present be seen as a technique in the screening of large potential target sets, rather than as an accurate predictive tool.

It is not fully understood which chemical traits of a molecule determine its solubility. Certain features, which are known to correlate well with solubility, e.g. LogP or melting point, need to be predicted or measured themselves, and cannot be read off of the construction of the molecule (like the number of atoms, for example). They may be easier and more accurate to predict than solubility, however, and there is certainly scope for such derived properties in solubility prediction. Delaney stated that a choice of LogP as a descriptor was obvious in his work [5], and the general solubility equation (GSE) was constructed on a theoretical basis using melting point as a parameter [10]. Both these models are able to predict solubility to the aforementioned accuracy.

The standard machine learning approaches, as well as our own, rely on a set of known descriptors, which are easily computed, for example through the online tool DRAGON [11], followed by supervised learning, for example neural networks.

In this paper, we analyse a publicly available data set found in [12] of drug-like molecules with regards to their solubility in water. We are interested in a more qualitative analysis of the features commonly associated with solubility prediction. In particular, we seek to understand which features, or combinations of features, might explain the solubility properties of the molecules.

### Main results

The long term aim of this work is to gain a qualitative understanding of the space of molecules. The premise underlying our work is that shape matters. In our context of solubility prediction, we consider two different concepts for shape: the space of molecules and the space of chemical data.

We study these using novel techniques from Topological Data Analysis (TDA), namely persistent homology and mapper (descriptions of both can be found in the Methods section), to tackle the issues connected to solubility prediction. There are two threads of analysis, one based on the mapper algorithm and the other based on persistent homology.

We use mapper to analyse chemical data space. We aim to understand the descriptors that affect solubility prediction and the interplay between them, instead of attempting to increase the accuracy of prediction, as we feel there is certainly potential for more explanatory model frameworks in this field.

Next, we use topological methods to create a measure of similarity between molecules that takes account of their physical shape, that is the 3D connectivity bond structure of the molecules.

In the first part, we investigate the feature space of the molecules using the mapper algorithm, where the features considered were calculated from SMILES strings. In the second part, we focus on the geometric shape of the molecules, which includes the positions and relative distances of the constituent atoms. We investigate this data using tools from persistent homology.

One significant feature that appears in our analyses is the number of atoms (nAT), which is closely linked with MW (molecular weight), a feature known to correlate well with solubility.

We find that another feature, nCIC (the number of cycles, or molecular rings), is an important shape descriptor. Both our threads of investigation agree on this conclusion.

It is known that chemically the number of cycles does affect solubility, fulfilling chemical heuristics, but the feature itself has not been used in the machine learning approaches to solubility prediction mentioned above.

We finally show how these topological features allow us to better understand the differences in properties that contribute to solubility.

### Related work

Persistence based methods have recently been used as a tool to discover new nanoporous materials [13], where they were used as an effective way to identify materials with similar pore geometries. Moreover, in a case study of materials for methane storage, it was shown that it is possible to find materials that perform as well as known top-performing materials by searching the database for materials with similar pore shapes. Conversely, the pore shapes of the top-performing materials can be sorted into topologically distinct classes, and materials from each class require a different optimisation strategy [13]. Furthermore, persistence has found use in a wide variety of materials applications, such as categorising amorphous solids [14], and analysing phase transitions [15, 16].

Persistent homology has also been used in the analysis of protein folding [17–20], and in particular persistent homology at different coarse-grained scales has been shown to enable the calculation of topological invariants in protein classes. Persistent homology has been used to relate molecular shape to binding affinity, and other molecular properties [21, 22]. Alternatively, persistent homology has been used as a descriptor in the construction of models of shape-dependent properties, such as in the case of fullerene stability [23].

In parallel, mapper based methods have found use in chemical fields. These range from the analysis of hyperspectral imaging data [24], to exploring protein folding pathways [25]. In these works, the mapper algorithm provides a visualisation technique for cluster analysis to detect minor compounds in a multiphase chemical system, and to detect low-density transient states in folding pathways, such as hairpins. Interestingly, standard computational chemistry analysis techniques have been introduced to understand structure in high-dimensional Euclidean data sets, such as in [26]. Here, the nudged elastic band algorithm, standard in determining minimum energy pathways, is used alongside Morse theory, as an alternative to both mapper and persistent homology.

## Materials and methods

In this section, we occasionally use standard mathematical terminology. Please refer to e.g. Chapters 1, 2 and Appendix 1 in [27] for the definitions of mathematical terms not explained in the text.

### Data set

The solubility measurements used in this study are those found in [12] and are collectively here referred to as the Wang data set. This data set contains several older datasets known to be reliable—Delaney [5], Huuskonen [4] and Solubility Challenge [7] among them. It contains 3663 molecules, given in Sybyl line notation (SLN) [28] form. Conversion from SLN to SMILES was performed using the RDKit python implementation [29].

The DRAGON [11] software suite was used for the calculation of molecular descriptors. It is important to note that using SMILES strings as input limits the descriptors that are calculated to one- and two-dimensional features. Such descriptors are quick to calculate but risk missing a complete description of chemical behaviour which depend on 3D properties. After some preprocessing (e.g. removal of constant descriptors) we had 1521 descriptors for the set of 3663 molecules via this approach.

When converting from SLN to SMILES, before calculating the descriptors, we lose isomeric information, such as chirality and cis-trans isomerism. We would expect the chiral compounds to have the same solubility, but the cis-trans ones probably do not. The descriptors we calculate actually do not depend on this isomerism. Although the different isomers are not duplicates originally, our methodology may cause some of them to appear as if they are. Running the subsequent mapper algorithm with and without these duplicates did not show any noticeable difference in the results, and we chose to include them in the data set. This robustness is a desirable property of the mapper framework.

**Fig. 1:**
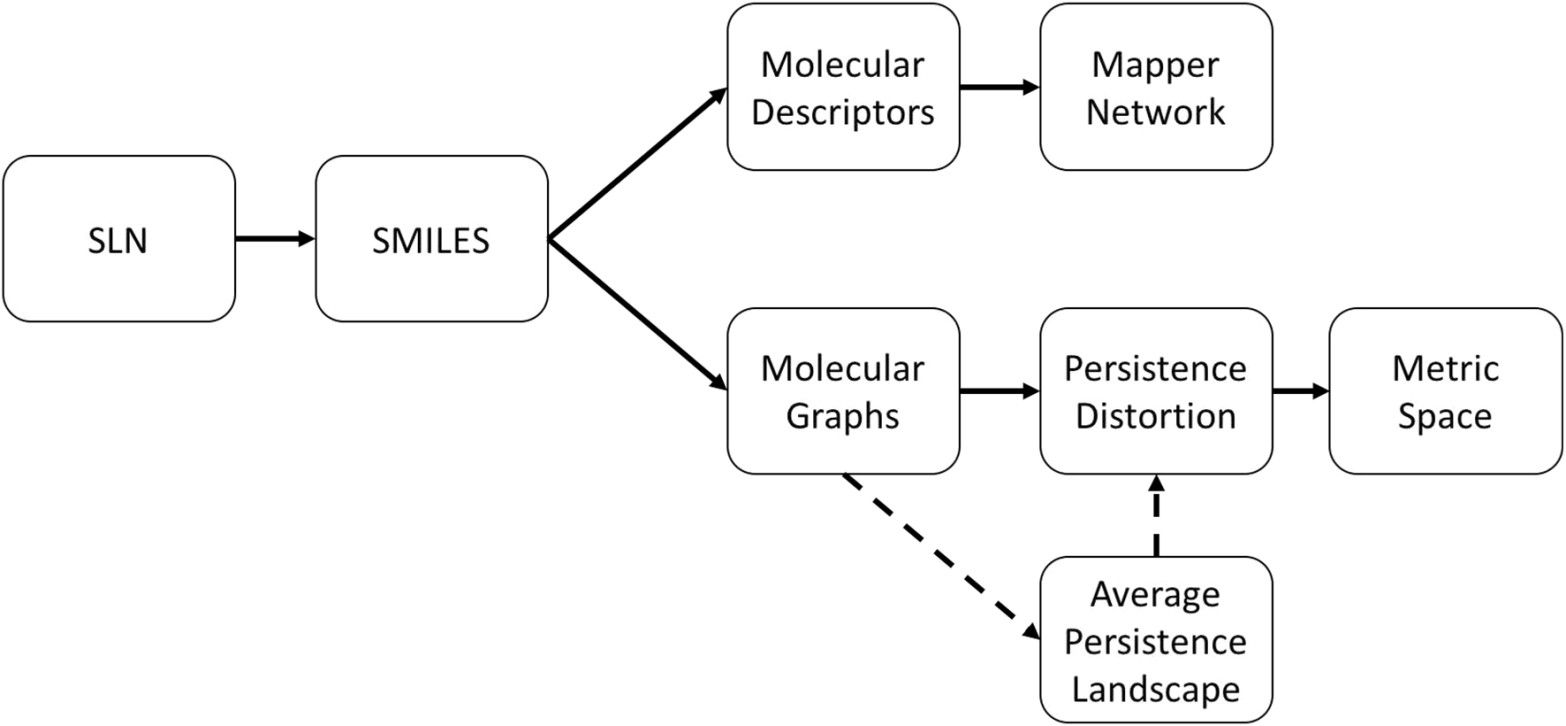
The pipeline. A flowchart illustrating the two main threads of study performed. Both the Mapper (top) and persistent homology (bottom) routes use simple molecular SMILES strings as input. We use dashed lines to emphasise that we use the average persistence landscape metric as a surrogate for the persistence distortion distance

The SMILES strings uniquely determine the molecular graphs. 3D atomic coordinates can be generated by using OpenBabel [30], to perform a classical geometry optimisation (using the MMFF94 forcefield). The 3D coordinates together with the bonds is considered to be a weighted, undirected graph, with weights defined by $L_2$ (or Euclidean) distances between the coordinates of the atom centres. We do not take into account bond order directly, however this is implicit in the bond lengths. A flowchart detailing our two main analytic pipelines can be seen in Fig. 1.

**Fig. 2:**
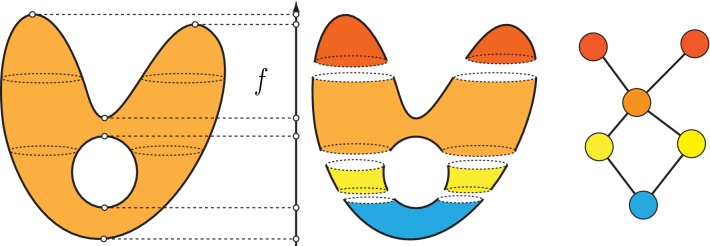
A continuous example of the implementation of the mapper algorithm, with the function *f* being the height and using the Euclidean metric

### The mapper algorithm

The Mapper algorithm is a method for visualising high-dimensional data and can be considered to be a generalisation of hierarchical clustering. A detailed description of the algorithm can be found in [31]. Here, we briefly summarise it.

The algorithm takes as input

- a data set *X*,
- a metric *d* on *X*,
- a scalar function $f:X\rightarrow {\mathbb {R}}$ and
- a covering by overlapping intervals $(a_i,b_i)$ of the image *f*(*X*).

The preimage of each interval, $f^{-1}(a_i,b_i)$, is clustered using hierarchical clustering, using *d* to measure the distance between data points. Next, a network is created whose vertices correspond to clusters and two clusters are connected by an edge if their intersection is nonempty. This creates a representation of data as a highly connected graph, as illustrated in Fig. 2.

**Fig. 3:**
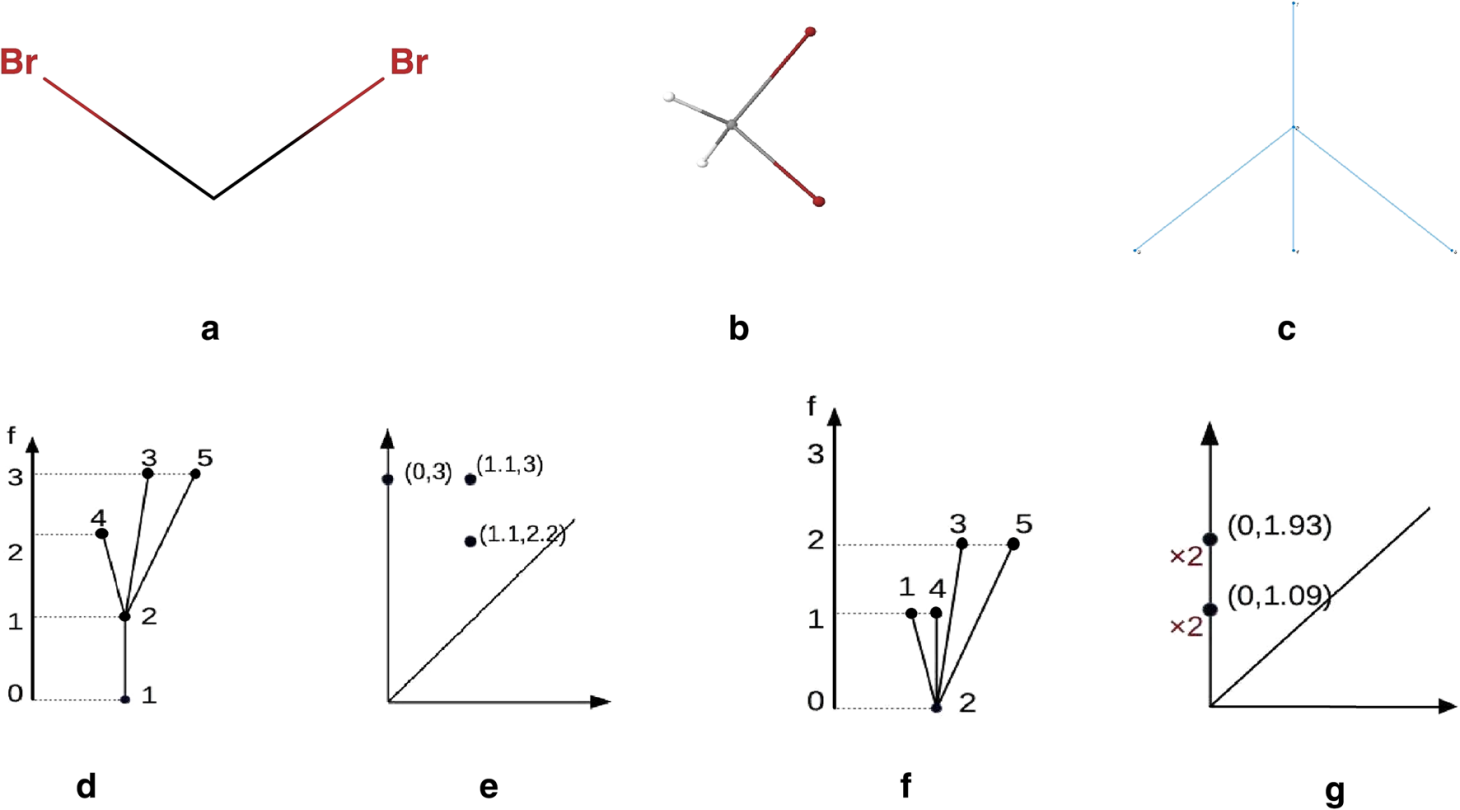
A visualisation of the superlevel set filtration of a molecular graph, for the molecule dibromomethane. The 3D model of the molecule **b** can be viewed as a metric graph (**c**). We first consider one of the hydrogen atoms as the base point (**d**), and get a corresponding persistence diagram (**e**). If, instead, we choose the carbon atom as the base point (**f**), we associate a different persistence diagram (**g**) to the graph. Note that in this second diagram the points have multiplicity 2

**Fig. 4:**
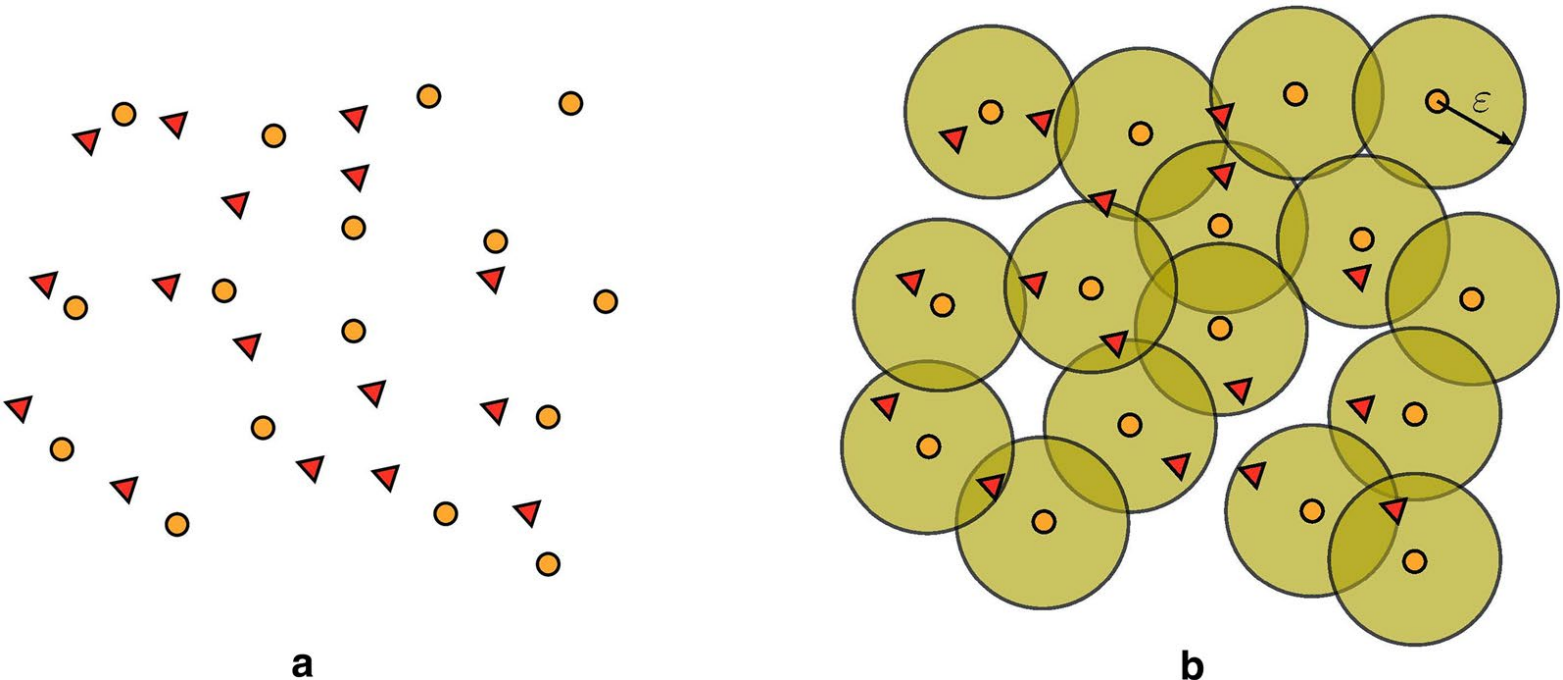
A visualisation of the Hausdorff distance. Let the red triangles be the subset *X*, and the orange circles be the subset *Y*. In **b**, the $\epsilon$ illustrates the $\sup _{y\in Y}\inf _{x\in X} d(x,y)$ part. It is the smallest number for which the disc of that radius around the orange circle farthest away from any of the triangles includes a triangle. To make the definition symmetric, this step is repeated for the triangles, and the maximum of the two radii is chosen

The most common approach to analyse these outputs when using mapper for feature detection, is to look for groupings of the data. We look for connected components within the mapper graphs, or find groupings of vertices that are highly connected, but more loosely connected to the rest of the structure. Afterwards, we analyse these groupings to find out which features best separate them from the rest of the data. Some of the more common metrics used in this approach are variations of the correlation and euclidean metric. We create the scalar function *f*, also referred to as the lens, using PCA, metric PCA (for metrics other than Euclidean), MDS and tSNE in the following way.

The PCA lenses generate a factorisation of the data matrix into linearly uncorrelated components. The first PCA lens is the coordinate given by the highest variance component, and the second corresponds to the second-highest variance. These lenses assume that the data supplied is using the Euclidean metric.

The metric PCA and MDS lenses compute a variant of the PCA coordinate lenses, for data that does not use the Euclidean metric. In the case of metric PCA, the data is first mapped into a Euclidean space using the rows of the distance matrix as the coordinates and then PCA is performed. Alternatively, MDS transforms the data into a Euclidean space, minimising the $L_2$ error. Both of these lenses therefore require distance matrices directly, rather than the coordinates.

The tSNE [32], or stochastic neighbour embedding, lenses generate an embedding of high-dimensional data into two dimensions by embedding a *k*-nearest neighbours graph of the data. A *k*-nearest neighbours graph is generated by connecting each point to its nearest neighbours.

### Implementation of mapper

In this paper we use an implementation of the mapper algorithm provided by Ayasdi. All of our analyses were created using Ayasdi Workbench.

It is possible to e a publicly available software, for example KeplerMapper [33], to reproduce our analyses. In KeplerMapper, the lenses (t-SNE, PCA) and metrics (called clusterer) are used via the *scikit* library. The covering is defined by the variables $n\_cubes$ and $perc\_overlap$.

In the Ayasdi implementation, the parameters *resolution*, *gain* and *equalize* manage the covering of the image by overlapping intervals. If the equalize parameter is set, the preimages of the intervals all contain the same number of data points. Resolution corresponds to the number of intervals the image *f*(*X*) is partitioned into, while gain corresponds to the amount of intersection between neighbouring intervals, i.e.

$$\begin{aligned} \text {percent of intersection} = 1-\frac{1}{\text {gain}}. \end{aligned}$$

Increasing the resolution will create a topological model that contains a larger number of nodes. Increasing the gain increases the number of edges in the network.

In this implementation, the tSNE lenses are referred to as the Neighbourhood lenses. The *k*-nearest neighbours graph is embedded in two dimensions using Ayasdi’s proprietary graph layout algorithm used in their visualisations. These lenses work to emphasise the metric structure of the data. Because these lenses are the *x* and *y* coordinates of this two-dimensional embedding, it is recommended using both of them together whenever one uses these lenses, and not to equalise them. The neighbourhood lenses use the selected metric to compute the lens.

In case one wishes to use the data without normalising it first, there are variations of the Euclidean metric and correlation, called *Variance Normalized Euclidean* and *Norm Correlation*, that take this into account.

### Persistent homology on graphs

The second thread of this work involves the use of persistent homology on the molecular graphs to create a measure of similarity. For this we use a variation of a distance between metric graphs proposed in [34].

We view molecules as simple, undirected graphs equipped with a weight function on the edges, which assigns a nonnegative number—the physical distance between the atom centres—to the edges (see Fig. 3). The weight function allows us to view the graph *G* as a metric space $(|G|,d_G )$, which allows the use of persistent homology. Here |*G*| is the set of points of this metric space, consisting of all the vertices of *G* together with the points of the edges considered as line segments of length equal to the weight assigned to the edge. For any two points $z,w\in |G|$, the distance $d_G(z,w)$ is given by the minimum length of a path connecting *z* to *w* in |*G*|.

**Fig. 5:**
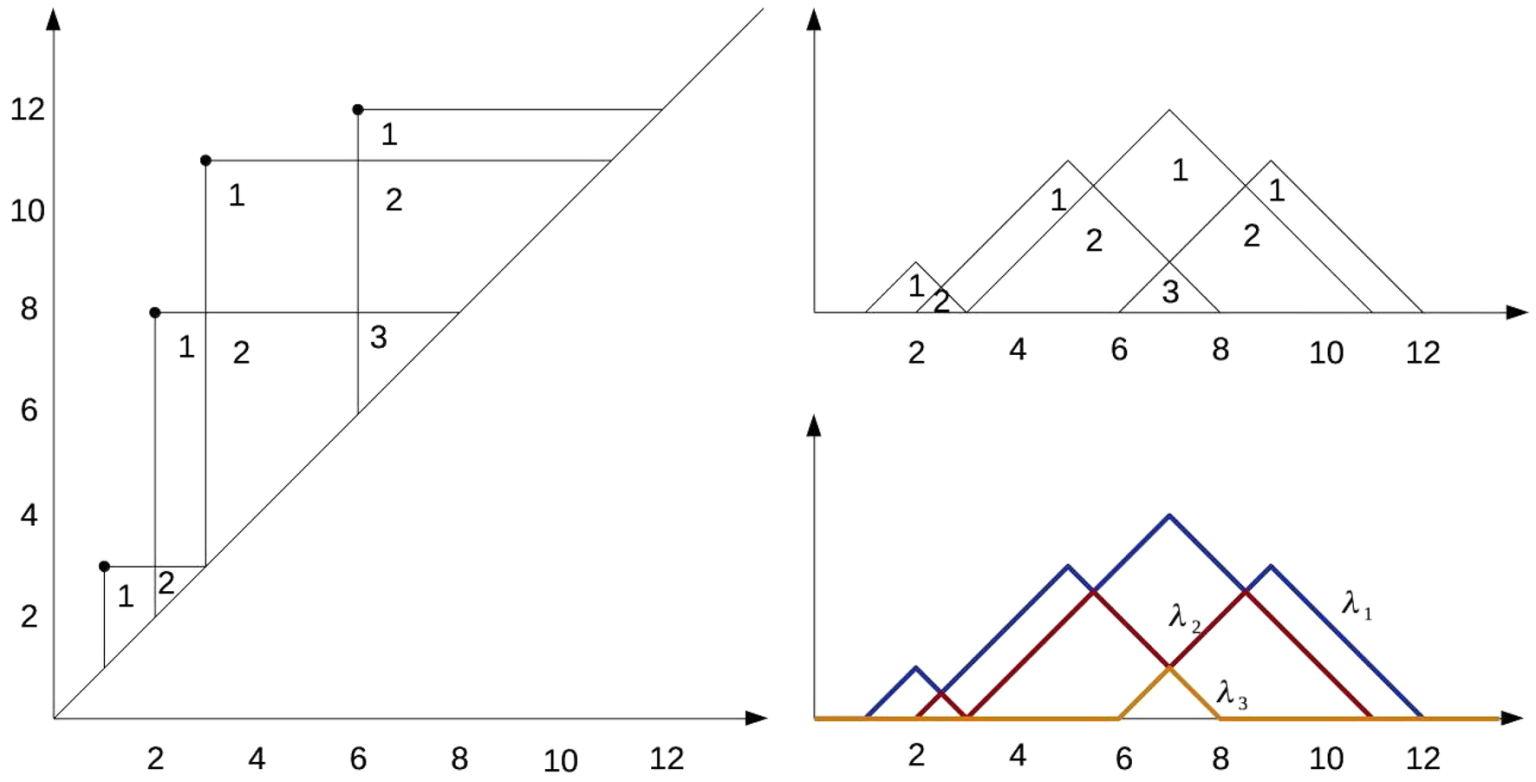
A visual explanation of persistence landscapes. The persistence diagram (left) is tilted, so that the diagonal becomes the new horizontal axis (top right). The $\lambda _i$ are the piecewise linear functions (bottom right)

In general, to a metric space (*X*, *d*) equipped with a function $f:X\rightarrow {\mathbb {R}}$ one can assign a persistence diagram as follows: First, we define the *super-level set of X* *with respect to *$\alpha \in {\mathbb {R}}$ by

$$\begin{aligned} X^\alpha :=\{x\in X \ | \ f(x)\ge \alpha \}. \end{aligned}$$

If $\alpha _1 > \alpha _2$ we have the inclusion $X^{\alpha _1}\subseteq X^{\alpha _2}$.

For $p=1,2,\ldots$, the *p*th homology gives information about the *p*-dimensional *holes*: For $p=0$, this refers to connected components, for $p=1$, it refers to loops, for $p=2$, it is cavities or voids, etc. The number $\alpha$ is called a *p*-critical value of *f* if the number of connected components of $X^{\alpha -\epsilon }$ and $X^{\alpha +\epsilon }$ changes for $p=0$, or if the number of loops in $X^{\alpha -\epsilon }$ and $X^{\alpha +\epsilon }$ changes for $p=1$, for all small $\epsilon >0$. For graphs, we do not calculate higher homologies (as graphs are 2 dimensional structures).

For metric graphs this works as follows. Fix a vertex *v* in *G*, and define a function $f:|G|\rightarrow {\mathbb {R}}$, called the height function at *v*, which assigns to each point $x\in |G|$ the path distance $d_G(v,x)$. Note that for general metric spaces *X*, it is also possible to consider *sub-level* sets of *X*, but in the case of metric graphs with the path distance, the number of connected components would always be the same (it would always equal 1). See Fig. 3 for a visual explanation. This is why it is more interesting to consider super-level sets for this particular height function.

**Fig. 6:**
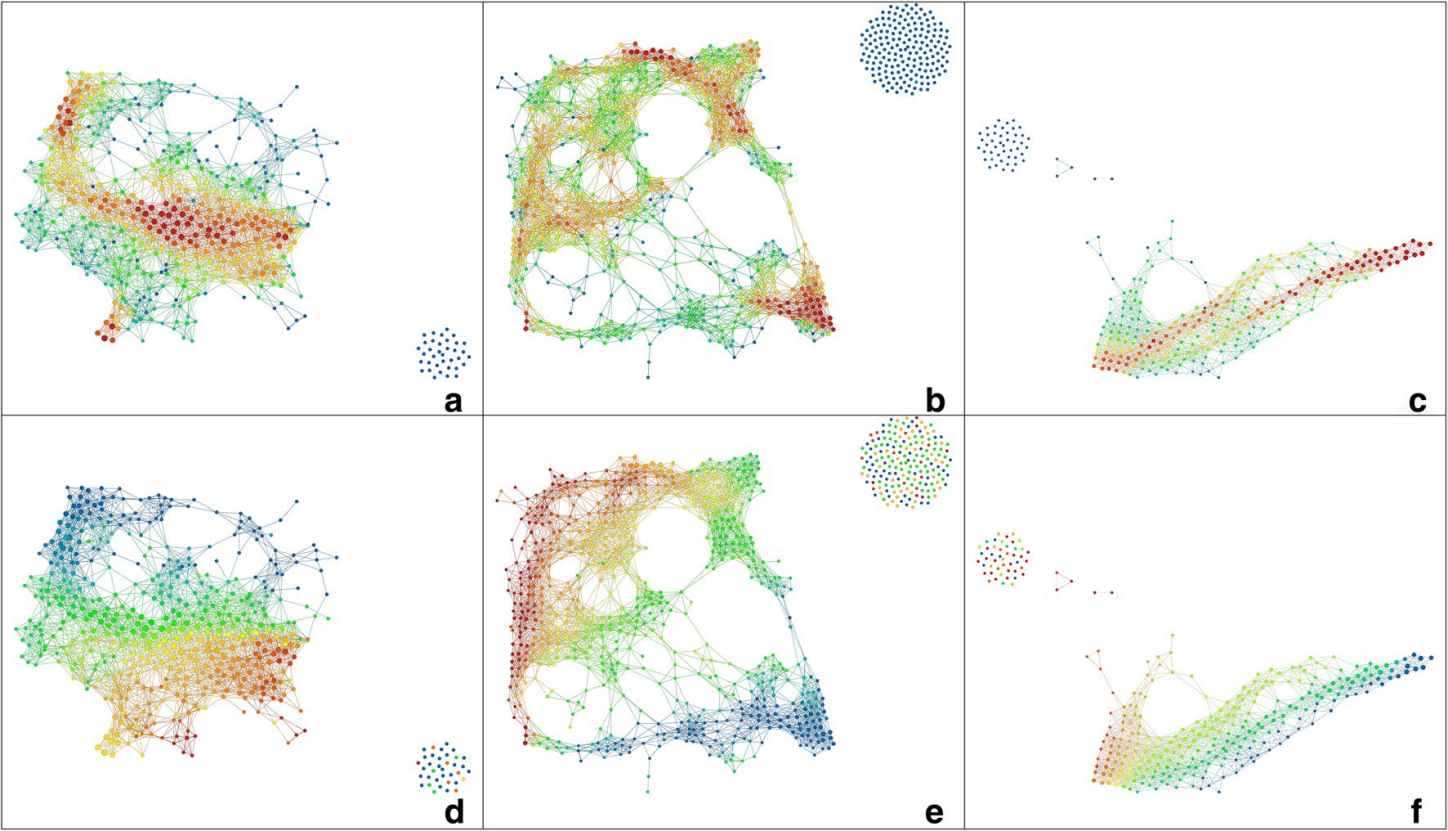
The first row shows three different analyses coloured by rows per node. The red patches indicate groupings of a large number of molecules. The first analysis uses the MDS lenses and norm correlation metric (resolution: 30, gain: 2.5, not equalized), the second is MDS lenses and Variance Normalized Euclidean metric (resolution: 35, gain:, 2.5, equalized) and the last one uses PCA lenses and the Variance Normalized Euclidean metric (resolution: 30, gain: 2.5, equalized). The second row shows the same analyses coloured by nCIC. Here blue corresponds to no cycles, green to 1 cycle, etc. The presented graphs have been created using Ayasdi Workbench

We take all *p*-critical values $\alpha _1>\alpha _2>\cdots >\alpha _n$. Then the super-level sets connected by natural inclusion maps give rise to a filtration:

$$\begin{aligned} X^{\alpha _1}\subseteq X^{\alpha _2}\subseteq \cdots \subseteq X^{\alpha _n}=X. \end{aligned}$$

The zeroth persistence diagram $Dg_0(f)$ captures the connected components that were born or died passing through a critical point. It consists of a set of points in the plane $\{(a,b)\in {\mathbb {R}}^2 \ | \ a<b\}$ (see Fig. 3e, g). Each point can occur more than once. The coordinates *a* and *b* of a point indicate the birth and death times of the connected components. The multiplicity of the point indicates the number of connected components that were born at time *a* and died at time *b*. The first persistent diagram $Dg_1(f)$ does the same for loops instead of connected components.

**Fig. 7:**
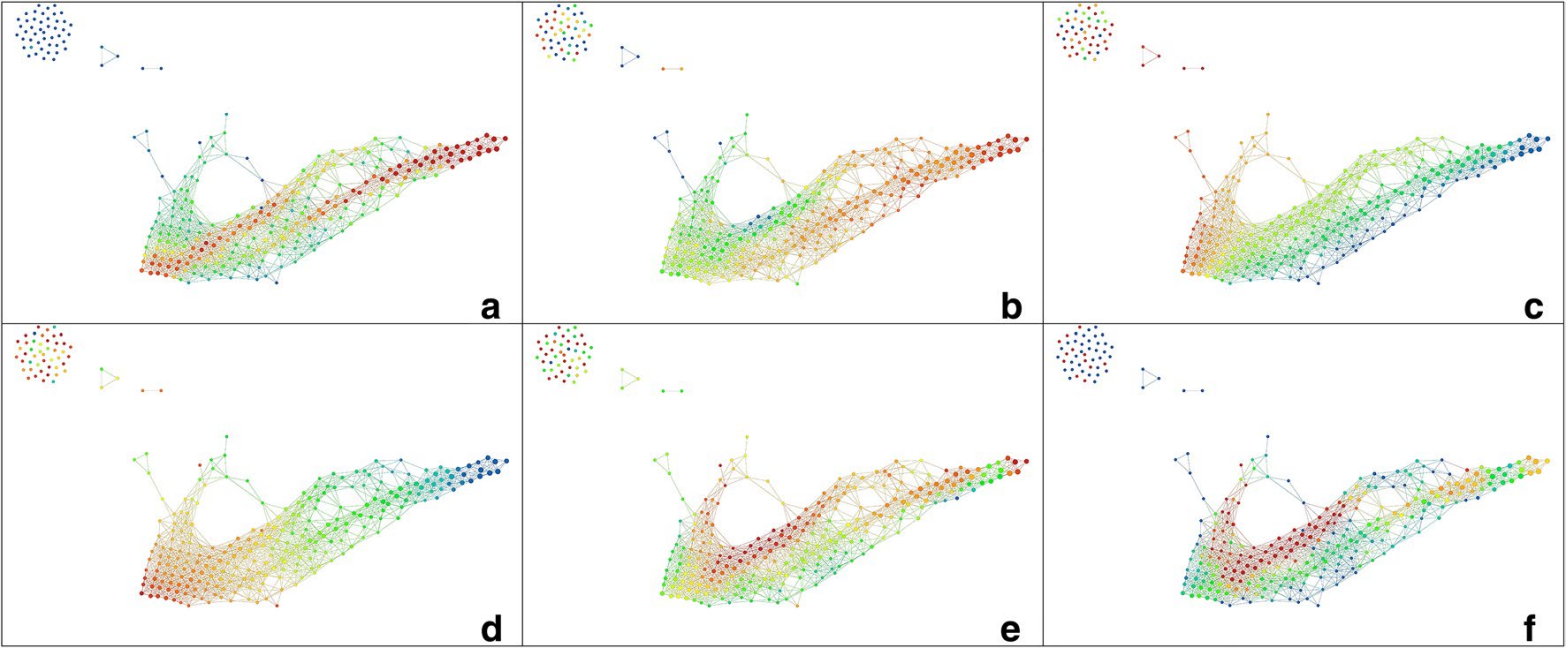
Coloured by rows per node (**a**), LogS (**b**), nCIC (**c**), MW (**d**), AMW (**e**) and nCL (**f**). We can see the red region in (**d**), corresponding to molecules with a high number of chlorines, matches the blue patch in (**b**). These are molecules with two rings, as we can see from (**c**), with a particularly low solubility. It is precisely these molecules which distort the colour gradient in (**b**). This visualisation was created using Ayasdi Workbench

A common measure of similarity of persistence diagrams is the *bottleneck distance*. It is stable with respect to perturbations of a filtration. It is the shortest distance $\delta$ for which there exists a perfect matching between the points of the two diagrams (where if there are different cardinalities, leftover points are mapped to the diagonal) such that any couple of matched points are at distance at most $\delta$. The set of persistence diagrams together with this metric can be considered as a metric space.

**Fig. 8:**
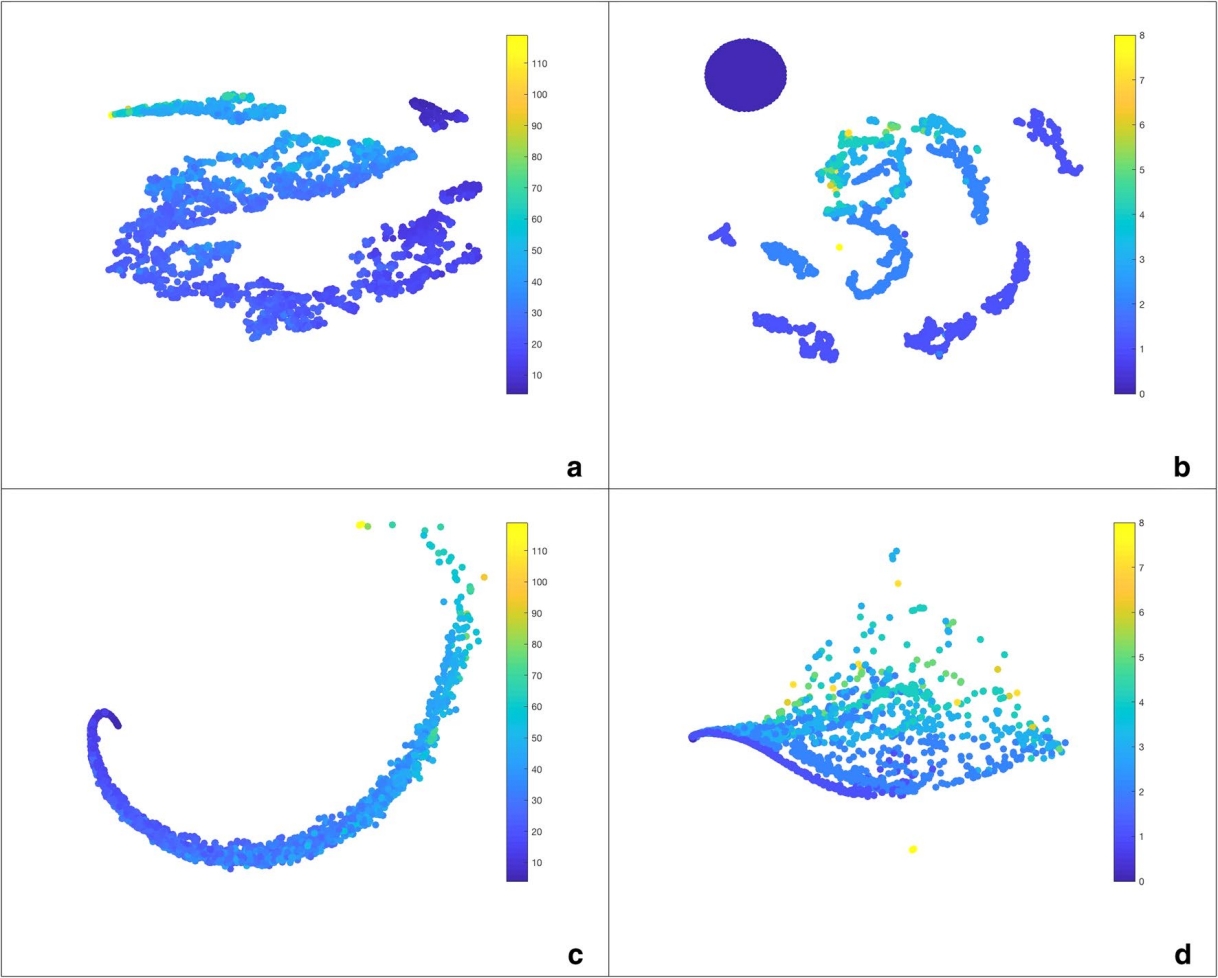
The first row shows tSNE embeddings of the $H_0$ (**a**) and $H_1$ (**b**) distance matrices, coloured by number of atoms and number of rings, respectively. The second row shows the MDS embeddings of the same

**Fig. 9:**
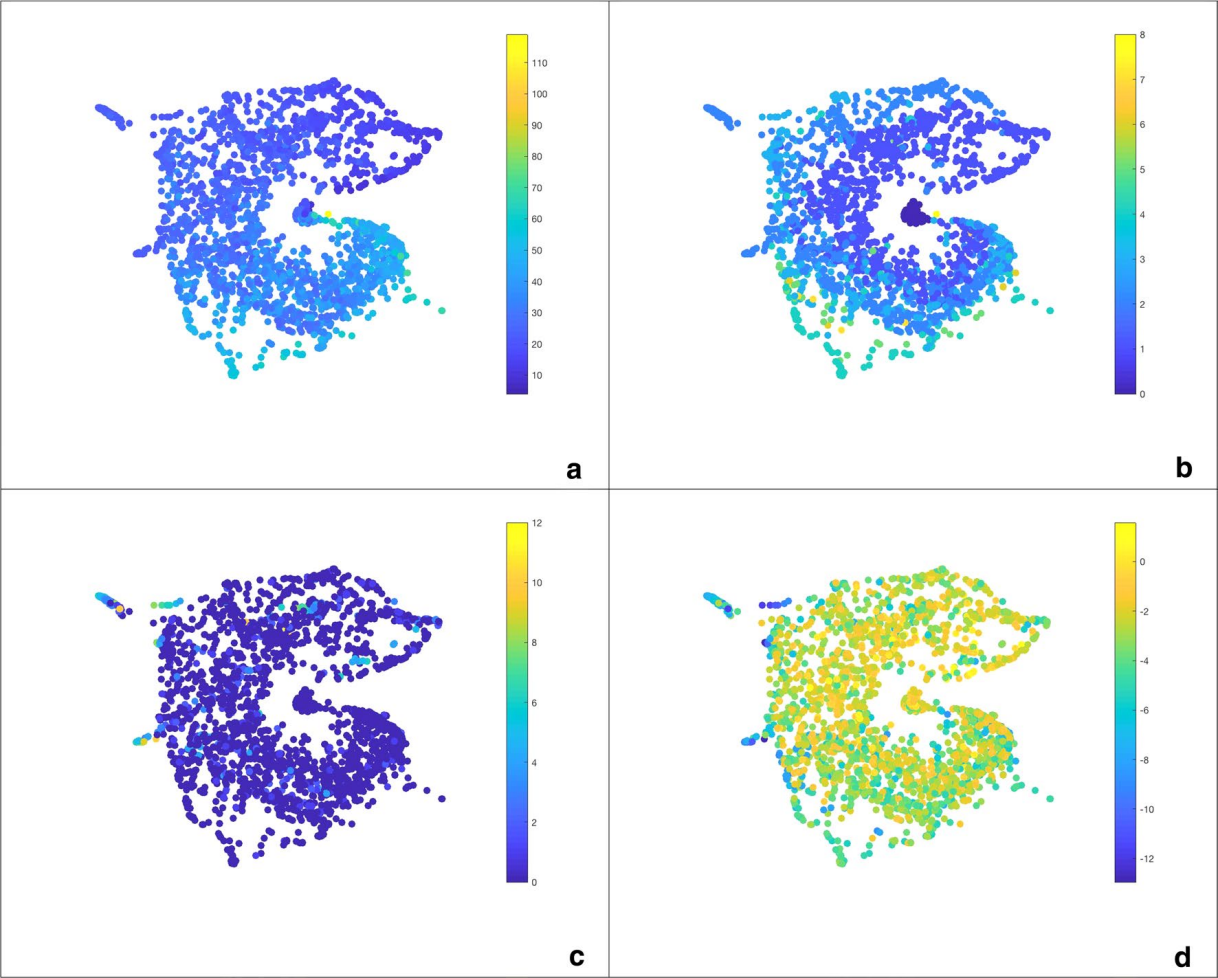
The tSNE planar embedding of the combined matrix constructed using SNF. Coloured by number of atoms (**a**), number of cycles (**b**), number of chlorines (**c**) and solubility (**d**)

In order to define the discrete PD distance, we make use of a more general construction, called the Hausdorff distance (illustrated in Fig. 4), which measures how far two subsets of a metric space are from each other. More precisely, for two non-empty subsets *X* and *Y* of a metric space (*M*, *d*), we define their Hausdorff distance by

$$\begin{aligned} d_H(X,Y)=\max \{ \sup _{x\in X}\inf _{y\in Y} d(x,y),\sup _{y\in Y}\inf _{x\in X} d(x,y) \}. \end{aligned}$$

For the definition of the discrete PD distance, only the 0-dimensional persistent homology is considered. Suppose we are given two metric graphs $(G_1,d_{G_1})$ and $(G_2, d_{G_2} )$. Let $(V_1, E_1)$ and $(V_2, E_2)$ denote the node and edge sets for $G_1$ and $G_2$, respectively.

Choose any vertex $s \in V_1$ as the base point, and consider the shortest path distance function $d_{G_1,s} : G_1\rightarrow {\mathbb {R}}$ defined as $d_{G_1,s}(x) = d_{G_1}(s,x)$ for any point $x \in G_1$. Let $P_s$ denote the 0-dimensional persistence diagram $D0(d_{G_1,s})$ induced by the function $d_{G_1,s}$. Define $d_{G_2,t}$ and $Q_t$ similarly for any base point $t\in V_2$ for the graph $G_2$.

This way, we associate a persistence diagram $P_s$ to each vertex $s\in V_1$, and similarly, a persistence diagram $Q_t$ is associated to each vertex $t\in V_2$. Because the persistence diagrams can vary for different vertices, we want to consider all possible ones, in order to end up with an invariant for the given graph. To the graph $G_1$ we associate the set of all persistence diagrams $C := \{ P_s | s\in V_1\}$. Similarly, to $G_2$, we associate the set $F := \{ Q_t | t \in V_2 \}$.

#### **Definition 1**

The discrete Persistence Distortion distance between $G_1$ and $G_2$, denoted by $d_{PD}(G_1,G_2)$, is the Hausdorff distance $d_H(C,F)$ between the two sets *C* and *F* where the distance between two persistence diagrams is measured by the bottleneck distance. In other words,

$$\begin{aligned} d_{PD}(G_1,G_2) = d_H(C,F). \end{aligned}$$

### Persistence landscapes

Persistence landscapes, first introduced by Bubenik [35], are an encoding of persistence diagrams by a sequence of continuous, piecewise linear functions (see Fig. 5). This allows statistics to be performed on them, the lack of which was a drawback of persistence diagrams. In particular, it is possible to calculate (unique) averages of landscapes. While the persistence landscape has a corresponding persistence diagram, the mean persistence landscape does not. The Landscapes toolbox [36] can translate persistence diagrams into landscapes, can compute averages of landscapes, $L_p$ distances and norms between landscapes, as well as the bottleneck distance.

### Implementation of graph persistence

For the calculation of the persistence diagrams on the molecular graphs, we use the rca1mfscm program, which is part of the TDAtools package developed by Harer et al. [37]. This program takes as input a simple, undirected graph *G* (without weights) with a function *f* defined on its vertices and edges. The requirement for the function *f* on *G* is that the values on the edges have to be greater than or equal to the values on the vertices. Such a function results in a filtration of *G* by sublevel sets of *f*.

In order to use this program to calculate an approximation of the Persistence Distortion distance, we need a superlevel filtration of the graph *G* by the path distance function starting at each choice of vertex.

For a choice of vertex *s*, let us consider the function *f* defined on vertices *v* by $f(v) = d_G(s, v)$ and on edges (*v*, *w*) by $f(v, w) = max\{ f(v), f(w) \}$. This is an approximation of the path distance function starting at *s*.

However, it is possible to use this program in our case, because there is the following relationship between superlevel and sublevel filtrations: the sublevel set filtration of a function *f* is the superlevel set filtration of the function $-f$.

So, while the values of *f* range from 0 at *s* to some maximum value $f(v_{max})$ at the (not necessarily unique) vertex $v_{max}$ farthest away from *s*, we define the filtration function *F* by an obvious reversal of *f*. That is, for $v\in V(G)$, we define

$$\begin{aligned} F(v) = max\{ f(v_i) \} - f(v) \end{aligned}$$

and $F(v,w) = max\{F(v),F(w)\}$. This function now has the value 0 at $v_{max}$ and the value $f(v_{max})$ at *s*. Then we use the rca1mfscm program to compute the sublevel set filtration of *F*. Afterwards, the persistence diagrams have to be ‘translated’ back.

As we repeat this process for each choice of vertex *s*, we end up with as many persistence diagrams as *G* has vertices.

In contrast to the PD distance, we computed persistence diagrams in both zero and one dimension. Computing the Hausdorff distance between the sets of persistence diagrams, in either dimension zero or one, proved too expensive for the number of molecules in our set. Instead, we use persistence landscapes to first average over all diagrams corresponding to the different vertices. Then we calculate the bottleneck distances between these average landscapes, ending with two distance matrices, one for $H_1$ and one for $H_0$. For visualisation and analysis, these can be embedded into lower dimensions using different dimensionality reduction methods, like MDS, PCA or tSNE.

### Similarity network fusion

Similarity Network Fusion (SNF) [38] is a recent computational method for data integration. Briefly, SNF combines many different types of measurements for a given set of samples. For *n* data points with *m* different types of measurements, *m* different $n\times n$ distance matrices are constructed, which can be thought of as a network on *n* points, with the distances being the weights on the edges. First, these are transformed into similarity matrices *W* by using an exponential similarity function. The SNF implementation takes these similarity matrices as input. To compute the fused matrix from multiple types of measurements, a full similarity matrix *P* and a sparse similarity matrix *S* are defined for each measurement. For the first, *P* is constructed by performing a form of normalisation on *W*, in the following way:

$$\begin{aligned} P(i,j) = \left\{ \begin{array}{ll} \frac{W(i,j)}{2\sum _{k\ne i} W(i,k)}, &{}\quad \text {for }j\ne i,\\ \frac{1}{2}, &{}\quad \text {for }j=i. \end{array}\right. \end{aligned}$$

The matrix *S* is constructed using *K* nearest neighbours. For each *i*, let $N_i$ represent the *K* nearest neighbours of *i*, including *i* itself, giving

$$\begin{aligned} S(i,j) = \left\{ \begin{array}{ll} \frac{W(i,j)}{\sum _{k\in N_i} W(i,k)}, &{}\quad \text {for }j\in N_i,\\ 0, &{}\quad \text {otherwise}. \end{array}\right. \end{aligned}$$

Next, the matrices *P* are iteratively updated to converge to a single similarity matrix. In the case $m=2$, the initial matrices are $P^{(1)}_{t=0} = P^{(1)}$, and $P^{(2)}_{t=0} = P^{(2)}$. The iterative step is given by

$$\begin{aligned} \begin{aligned} P^{(1)}_{t+1} = S^{(1)} P^{(2)}_{t} (S^{(1)})^T\\ P^{(2)}_{t+1} = S^{(2)} P^{(1)}_{t} (S^{(2)})^T. \end{aligned} \end{aligned}$$

After *t* steps, the overall status matrix is computed as

$$\begin{aligned} P^{(c)} = \frac{P^{(1)}_{t} + P^{(2)}_{t}}{2}. \end{aligned}$$

We transform our $H_0$ and $H_1$ distance matrices into similarity matrices using the same exponential function described above and then use SNF to combine them into one matrix.

## Results and discussion

### Feature discovery through mapper

The most straightforward feature selection or reduction technique is to simply see which features correlate best with the log of solubility feature. As solubility itself is a continuous feature, it is straightforward to use data analysis methods that are designed for this data type, however this approach excludes many potentially useful descriptors which happen to be discrete/categorical or binary/logical. As these are different data types, different statistics are calculated for them, and mixing data types and the different permissible statistics defined for them can be challenging.

This constraint does not apply to mapper. In fact, we found that one categorical descriptor, in particular, called nCIC in the data set, might give important information about how the shape of the molecules influences their solubility.

We performed several analyses using different metrics and lenses to discover embeddings of the data set that were grouping the data by solubility. We found that not normalising data and using the norm correlation and variance normalised Euclidean metrics gave better results. Several embeddings using different lenses showed a marked gradient when coloured by solubility.

After analysing these embeddings, we discovered that the same feature, nCIC (the number of cycles, or molecular rings), accounted best for the formation of cluster-like groupings within the output graphs. This remained consistent when changing between lenses and metrics, as well as varying the parameters of resolution, gain and equalisation in Ayasdi Workbench.

The feature nCIC is the one that according to mapper determines most strongly the similarity between molecules. This creates one possible depiction of chemical space. To see whether this measure of similarity is useful, we decided to partition our data set according to the number of cycles. Next, we looked at how the correlation values had changed within these subgroups.

**Table 1:** The table shows the changes in correlation values with solubility for the feature $X\%$, depending on the number of rings

nCIC	X%	AMW	MW
All	$-$ 0.3327	$-$ 0.2673	$-$ 0.5520
0	$-$ *0.0793*	$-$ *0.0392*	$-$ 0.4237
1	$-$ 0.3830	$-$ 0.3147	$-$ 0.5176
2	$-$ ***0.8163***	$-$ ***0.7203***	$-$ 0.5326
$>\,2$	$-$ 0.4318	$-$ 0.4024	$-$ *0.0213*
$\ne \,0$	$-$ 0.5458	$-$ 0.4311	$-$ 0.4954

Responsible for this change are the number of chlorine atoms in the molecule. Also shown are the correlation values of average molecular weight, which itself correlates well with $X\%$, and molecular weight. The highest (bolditalic) and lowest (italic) correlation values are emphasised

And indeed, we could make the following interesting observation: The feature that changes the most is $X\%$, the percentage of halogen atoms. In Table 1, we can see that for the whole set, its correlation with solubility is $-\,0.3327$. However, restricted to molecules with two rings we get a correlation of $-\,0.8163$. In molecular drug design, a large proportion of halogens tend to be chlorines, due to the relative ease of chlorination. We therefore chose to investigate nCL, the number of chlorines, to observe if it was this property that was affecting molecular solubility. Looking carefully at the makeup of the molecules in question, we were able to deduce that it is indeed the number of chlorine atoms (once again, a discrete feature), that is responsible for the increase of the percentage.

Molecular weight (MW), a feature known to correlate well with solubility, also shows some interesting beha viour, depending on the number of cycles. Overall correlation of MW with LogS is $-\,0.5520$. However, for molecules with more than 2 rings ($nCIC>2$), we get a correlation of $-\,0.0213$. This interesting behaviour of molecular weight versus average molecular weight becomes immediately visually apparent in the mapper graphs.

The embeddings have different shapes. However, they all agree on certain observations. We discuss the analysis of the PCA lens (Fig. 6c, f) in more detail.

In Fig. 7, we can see this analysis coloured by different relevant features. It has several distinct groupings of nodes (a). The red colour indicates a higher number of molecules per node. Coloured by solubility (b), we can see a gradient, as the average solubility values decrease from left to right. To investigate what gathers together the molecules in each cluster, we create subgroups of the data and using Ayasdi’s own tools, investigate what separates these subsets from the rest. The feature that seems to separate the groupings best is the number of cycles in the molecular graph, as can be seen in the third image (c). Finally, to compare, we show the same graph coloured by molecular weight (d). As a feature known to correlate with many other features, including solubility, it is to be expected that the mapper analysis will pick up on this. Indeed, a colour gradient can be observed, but it is not as obvious as in the case of the number of cycles.

### Applying persistent homology to molecular graphs

We have seen that molecules can be thought of as metric graphs. The metric, given by the path distance, turns these graphs into metric spaces, and more generally, into topological spaces.

Persistent homology of a topological space—a metric graph *G*, in our case—gives the topological invariants of *G* summarised in a persistence diagram. These invariants are used to distinguish topological spaces by means of the bottleneck distance which provides a pseudometric on the persistence diagrams or landscapes. This way we turn the set of molecular graphs into a pseudometric space, which is typically high dimensional.

To provide a visualisation of this molecular space, we use low-dimensional embeddings of the $H_0$ and $H_1$ distance matrices.

Using one of the low-dimensional embeddings discussed above, e.g. tSNE or MDS, the $H_0$ distance matrix shows a strong gradient when coloured by the number of atoms, or molecular weight, while the $H_1$ distance matrix shows a gradient when coloured by nCIC, as can be seen in Fig. 8.

It is interesting to note that the mapper algorithm points to the same topological descriptors of the molecules as persistent homology.

Using SNF, we combined the two distance matrices to get a single similarity matrix. This gives us a unified homology-based depiction of the space of molecular graphs. The tSNE embedding can be seen in Fig. 9. It retains the main characteristics of its components, showing a radial gradient with respect to the number of rings, and an angular gradient with respect to the number of atoms. When coloured by the number of chlorines, we can see that the small, distinct subsets in the upper left corner correspond to the molecules with two rings which we found using mapper. While they also appear in this depiction of chemical space, it is on the whole not as intuitive to interpret and draw conclusions from as mapper networks.

### Combining persistence and mapper

Using the persistence landscapes toolbox, we can compute the $L_p$ norms of persistence landscapes. In our case, we computed $L_2$ norms. This way we obtain two new features (one for the $H_0$ persistence diagrams and one for the $H_1$ persistence diagrams). These feature vectors contain continuous values. In effect, this is a way to create related, continuous features for the discrete variables nAT and nCIC. Continuous variables are in general preferable to discrete ones, as there are a wider variety of options available for their analysis (most notably correlation). The newly computed variables can also be added to the data matrix to be input into Ayasdi Workbench where they give very similar results to their discrete counterparts.

## Conclusion

We performed a systematic study of in silico calculation of aqueous solubility of molecules utilizing the methodology of topological data analysis.

TDA provided molecular-scale understanding of how the ring structure of the molecules affects solubility. In particular, TDA naturally allows us to see how the impact of chlorine affects the variation of solubility as a function of ring count.

While our analyses do not provide a quantitative prediction of solubility, this approach illustrates how minor changes in molecular design affect the physical properties of the bulk.

We have used techniques from topological data analysis, namely mapper and persistent homology, to understand chemical space and also to aid in solubility prediction. Mapper provided useful insights into the structure of a descriptor space generated by a standard cheminformatics software, and made subtle correlations far more prominent. In particular, it was seen that the effect of chlorinated groups to reduce solubility was far more powerful in larger molecules in our data set. This behavior is clear from the mapper output, even though the vast majority of molecules have no chlorines. Furthermore, the molecules with chlorinated groups are evenly distributed as a function of the number of rings. We are therefore confident that this is a real effect, and not a product of our data set.

Persistent homology allowed the determination of a chemical shape space, through the persistence distortion distance on weighted chemical graphs. Using this dissimilarity, we were able to produce a set of metric shape spaces. Using different degrees of homology, we were able to separate molecules both by their atom numbers, and their ring counts. Understanding the structure inside these groups is an area of further study.

Furthermore, we were able to use norms on persistence landscapes to convert these discrete descriptors into continuous ones. In particular, we envisage the continuous analogue of ring count will prove a useful descriptor in traditional QSPR approaches, as now quantities such as correlation will have more meaning.

This graphical depiction of chemical space might provide use in the field of chemography. In particular, we envisage the use of such tools in projects such as ‘The Chemical Space Project’ [39], or an alternative to traditional topographic mapping, such as in [40]. Topological networks, as output by the mapper algorithm, could also provide an alternative to current chemical exploration tools, such as Pharmit [41].

## Additional files

**Additional file 1.** The Wang dataset of the molecules analysed, with the descriptors used in the mapper analysis.

**Additional file 2.** The pairwise distance matrix of the zero-dimensional persistence diagrams.

**Additional file 3.** The pairwise distance matrix of the one-dimensional persistence diagrams.

**Additional file 4.** The combined similarity matrix of the distance matrices Additional file 2 and Additional file 3, using SNF.

**Additional file 5.** The MATLAB program to compute the persistence diagrams for the molecules using the TDATools software.

## Footnotes

1 This refers to solubility as measured in molar concentrations, and Log base 10 as will be standard throughout the text.
